# X-exome sequencing in Finnish families with Intellectual Disability - four novel mutations and two novel syndromic phenotypes

**DOI:** 10.1186/1750-1172-9-49

**Published:** 2014-04-11

**Authors:** Anju K Philips, Auli Sirén, Kristiina Avela, Mirja Somer, Maarit Peippo, Minna Ahvenainen, Fatma Doagu, Maria Arvio, Helena Kääriäinen, Hilde Van Esch, Guy Froyen, Stefan A Haas, Hao Hu, Vera M Kalscheuer, Irma Järvelä

**Affiliations:** 1 Department of Medical Genetics, Haartman Institute, University of Helsinki, Helsinki, Finland; 2 Outpatient Clinic for Persons with Intellectual Disabilities, Tampere University Hospital, Tampere, Finland; 3 Norio Centre, Department of Medical Genetics, Helsinki, Finland; 4 Department of Clinical Genetics, Helsinki University Central Hospital, Helsinki, Finland; 5 Child Neurology, Päijät-Häme Central Hospital, Lahti, Finland; 6 National Institute for Health and Welfare, Helsinki, Finland; 7 Center for Human Genetics, University Hospital Leuven, Leuven, Belgium; 8 Human Genome Laboratory, Department of Human Genetics, VIB Center for the Biology of Disease, KU Leuven, Leuven, Belgium; 9 Department of Computational Molecular Biology, Max Planck Institute for Molecular Genetics, Berlin, Germany; 10 Department of Human Molecular Genetics, Max Planck Institute for Molecular Genetics, Berlin, Germany

## Abstract

**Background:**

X-linked intellectual disability (XLID) is a group of genetically heterogeneous disorders characterized by substantial impairment in cognitive abilities, social and behavioral adaptive skills. Next generation sequencing technologies have become a powerful approach for identifying molecular gene mutations relevant for diagnosis.

**Methods & objectives:**

Enrichment of X-chromosome specific exons and massively parallel sequencing was performed for identifying the causative mutations in 14 Finnish families, each of them having several males affected with intellectual disability of unknown cause.

**Results:**

We found four novel mutations in known XLID genes. Two mutations; one previously reported missense mutation (c.1111C > T), and one novel frameshift mutation (c. 990_991insGCTGC) were identified in *SLC16A2*, a gene that has been linked to Allan-Herndon-Dudley syndrome (AHDS). One novel missense mutation (c.1888G > C) was found in *GRIA3* and two novel splice donor site mutations (c.357 + 1G > C and c.985 + 1G > C) were identified in the *DLG3* gene. One missense mutation (c.1321C > T) was identified in the candidate gene *ZMYM3* in three affected males with a previously unrecognized syndrome characterized by unique facial features, aortic stenosis and hypospadia was detected. All of the identified mutations segregated in the corresponding families and were absent in > 100 Finnish controls and in the publicly available databases. In addition, a previously reported benign variant (c.877G > A) in *SYP* was identified in a large family with nine affected males in three generations, who have a syndromic phenotype.

**Conclusions:**

All of the mutations found in this study are being reported for the first time in Finnish families with several affected male patients whose etiological diagnoses have remained unknown to us, in some families, for more than 30 years. This study illustrates the impact of X-exome sequencing to identify rare gene mutations and the challenges of interpreting the results. Further functional studies are required to confirm the cause of the syndromic phenotypes associated with *ZMYM3* and *SYP* in this study.

## Background

Intellectual disability (ID) can be defined as a significant impairment of cognitive and adaptive functions and has an estimated prevalence of 1.5-2% [1]. It is defined by an intelligence quotient (IQ) below 70 and an impairment in social and adaptive skills diagnosed before the age of 18 years [2]. ID can be caused by genetic as well as Anon-genetic factors. A consistent finding among individuals with ID has been the excess of males [1], indicating a role of defects on the X-chromosome. Mutations in X-linked genes contribute for 10%-15% of ID cases in males [3]. There is substantial evidence that X-linked ID (XLID) is genetically an extremely heterogeneous disorder [4].

The recent development of sequencing technologies has provided an effective tool to analyze the chromosomal distribution of mutations underlying ID [5-10]. Sequencing of the coding regions of the X-chromosome in families with multiple affected males has resulted in the identification of about 100 XLID genes to date [11]. Also in sporadic male patients, the possibility of a *de novo* mutation on the X-chromosome has to be taken into consideration [12].

The aim of this study was to identify the causative gene defects underlying XLID in Finnish families with two or more affected males.

## Methods

### Patients

Families with two or more affected males with ID were enrolled in this study (n = 14). Many of these patients have been under the care of experienced child neurologists and/or clinical geneticists since 1980. Inspite of detailed clinical investigations the diagnosis for these families has remained unknown. The Ethical Committee of the Helsinki University Central Hospital approved this study. Written informed consent was obtained from either the parents or the guardians of the patients. DNAs from Finnish blood donors were used as controls.

### Exome sequencing

For enrichment of genomic DNA from 11 index patients we used the Agilent SureSelect Human X-Chromosome Exome kit (Agilent, California, USA) and for three index patients (D299, D301, D303) we performed droplet-based multiplex PCR (7,367 amplicons, 757 genes, 1.54 Mb) similarly to the previously described study [13]. DNAs were sequenced on the Illumina GAII and HiSeq2000 platforms, respectively. For all samples coverage of at least 10X was reached for >86% of the targeted regions. Average sequencing depth ranged between 149–583. The sequence files were then aligned against the hg19 reference genome by SOAP2.21 with the default settings. Variants in the form of SNVs, indels and CNVs were called by an in house software package (Medical Re-sequencing Analysis Pipeline (MERAP), developed at the Max Planck Institute for Molecular Genetics, Berlin. All sequence variants were screened against publicly available databases (dbSNP138, 1000 Genomes project, Exome Variant Server (ESP6500) and the in-house database of the Max Planck Institute, Berlin) for annotating likely non-pathogenic and previously reported neutral variants. In addition, the OMIM catalogue and the Human Gene Mutation Database (HGMD) were used as a filter to identify all previously described pathogenic changes.

### Mutation analysis and Sanger sequencing

Validation of variants was performed using the standard Sanger sequencing protocol. First, primers (Oligomer Ltd, Helsinki, Finland) were designed to detect each individual mutation using their respective reference sequence. All PCR reactions were performed in 25 μl volume using Dynazyme polymerase (Finnzymes, Espoo, Finland) under standard conditions. PCR products were purified using Exo-Sap^IT^ (Affymetrix, Santa Clara, CA) according to the standard protocol. Sequencing reactions were performed using Applied Biosystems BigDye terminator v3.1 (Life Technologies, Carlsbad, CA) and products were analyzed on the ABI 3730 sequencer. Finally, the accurate genotype of each variant was confirmed by sequence analysis with the Sequencher software v4.8 (Life Technologies) in both the family members and the anonymous Finnish controls. Primer sequences are available on request.

### X-inactivation analysis

X-inactivation study was performed in the families with symptomatic carrier females. 500 ng of DNA was divided into two fractions; one set was digested in a 20 μl volume of buffered solution with *Hpa*II (New England Biolabs, UK) while the other set was digested with *Rsa*I (Roche, Basel, Switzerland). Both aliquots were incubated at 37°C for 17 hours. Next, exon 1 of the androgen receptor gene (*AR*) was amplified from 2 μl of each of the *Hpa*II and *Rsa*I digested DNA samples using the forward 5'-labelled primer TCCAGAATCTGTTCCAGAGCGTGC and CTGGGACGCAACCTCTCTC reverse primer [14]. The amplification was performed for 35 cycles with an annealing temperature of 63.3°C. DNAs from healthy males were used as controls. Two μl of the PCR product was mixed with 10 μl of Hi-Di formamide (Life Technologies) and 0.025 μl of GeneScan 500 Liz size marker (Life Technologies). The samples were run on an ABI3730xl genetic analyzer. The peaks were analyzed using the GeneMarker software version1.4 (Life Technologies).

### *In silico* analysis

Multiple programs, including SIFT, PolyPhen-2, PhD-SNP, PANTHER and SNPs&GO were used to predict the functional impact of the mutation on the protein function [15-19].

Multiple protein sequence alignment using orthologous species and human sequences was performed using ClustalX to verify the evolutionary conservation of the respective amino acid positions [20]. The HOPE server was used to predict the structural effects of the mutation [21]. In addition, we used the ConSeq server to analyze the evolutionary conservation of the structures of proteins [22].

## Results

Computational analysis of the sequencing data revealed a total of 1,032 variants in coding and non-coding exons on chromosome X, or potentially affecting splice sites. Out of these, 405 protein changing mutations were either present in publicly available databases or were recurrent in our in-house dataset. The final set of 25 novel variants including those that were reported as heterozygous in publicly available databases, the recurrent pathogenic *SLC16A2* mutation present in HGMD and the recurrent variants investigated for segregation in this study are listed in the Additional file 1: Table S1. In four families, D299, D174, D172 and D301 a novel mutation in an X-chromosomal gene was found to co-segregate with the clinical phenotype. In one family L107, a recurrent pathogenic mutation in an X chromosomal gene was identified. In one family D222, a novel missense mutation in a candidate XLID gene was identified. The clinical phenotypes and the identified mutations are described below. In the remaining eight families no functionally relevant candidate genes were found.

### Clinical description of the patients and X-exome sequencing results

#### Family L107

**Figure 1:**
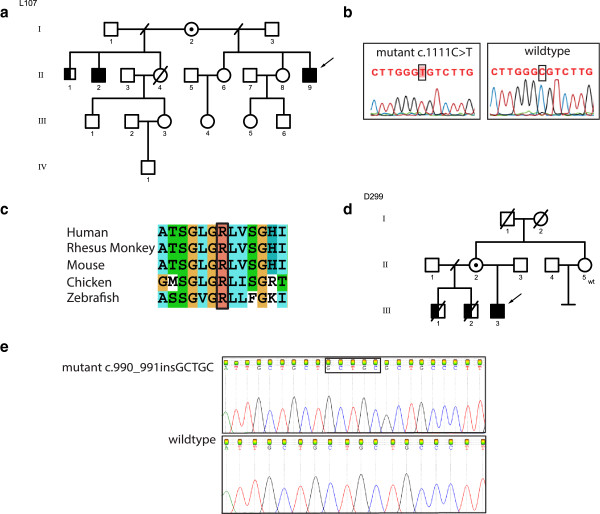
**Overview of the mutations reported in *SLC16A2 *gene in two Finnish families with Allan Herndon Dudley Syndrome. a)** Family pedigree of L107 showing the inheritance of *SLC16A2* mutation, open circles denote females; circles with a dot in the middle denote obligate carrier females, empty square denote males, the left half of the black squares denote affected males, the right half of the squares denote mutation positive males, crossed symbols denote deceased individuals, wt denote mutation negative subject, **b)** Sanger sequencing confirming the missense mutation c.1111C > T, **c)** Multiple species protein sequence alignment showing conservation of the mutated R371 residue, **d)** Family pedigree of D299 showing the inheritance of *SLC16A2* mutation, **e)** Sanger sequencing confirming the frameshift insertion c.990_991insGCTGC.

Family L107 has three affected males (II-1, II-2 and II-9, Figure 1a), who presented with moderate to severe ID. Patients II-1 born in 1950 and II-2 born in 1954 never walked. They have spastic diplegia in the lower limbs, athetosis in the upper limbs, alalia and dysarthria. In contrast, the index patient (II-9) born in 1966 who is the half-brother could walk but lost his motor skills during early childhood. Patients II-2 and II-9 could speak a few words. No data was available from patient II-1 concerning speech. Clinical features are summarized in Table 1. Metabolic analyses, EEG, EMG and imaging were normal. The thyroid hormone levels for TSH and T_4_ from the index patient II-9 were within the normal range. Data about the thyroid hormone level for T_3_ was unavailable. The index patient (II-9) was previously analyzed for *MECP2* aberration, as well as for mutations in *ARX* and *CDKL5*, all being normal. High-resolution, X-chromosome specific arrayCGH was also reported to be normal. X-exome sequencing of the index patient (II-9) identified a variant in *SLC16A2*, *BCORL1* and *GPR112*, respectively. Variants in *BC0RL1* and *GPR112* were excluded as they were not clinically relevant. A missense mutation [NM_006517.4:c.1111C > T; p.Arg371Cys] in exon 4 of the *SLC16A2* gene was identified and was compatible with the clinical phenotype. This mutation was also present in his half-brother (II-2) and his mother (I-2) was found to be carrier of this mutation. The oldest affected half-brother (II-1) did not participate in the mutation analysis study. According to PolyPhen-2, SIFT, PhD-SNP, SNPS&GO, and PANTHER, the mutation is predicted to be damaging. The ConSeq server also revealed that this residue is highly conserved with a score of 9. Based on HOPE [21], the altered amino acid has a change in charge and can disturb the ionic interactions with the other transmembrane helices. The mutant cysteine residue is more hydrophobic than the wild type arginine residue, possibly affecting the hydrophobic interactions within the core of the protein or with the membrane lipids and, as a result, disturbing the correct folding of the protein. The mutation was absent in 100 Finnish controls.

**Table 1:** Overview of the clinical features of the previous and present families with Allan-Herndon-Dudley syndrome

**Clinical findings**	**Clinical findings reported previously**	**D299**	**L107**
**Short stature**	+	+	-
**Scoliosis**	+	+	+
**Low weight**	+	+	+
**Microcephaly**	+	+	**-**
**Muscle hypoplasia**	+	+	NA
**Hypotonia**	+	+	NA
**Contractures**	+	+	NA
**Dystonic movements**	+	+	+
**Athetosis**	+	+	+
**Intellectual disability**	+	+/S	+/MO/S
**Absent speech**	+	+	+
**Dysarthria/Limited speech**	+	+	+
**Seizures**	+	+	NA
**Pectus excavatum**	+	+	NA
**Narrow long face**	+	-	+
**Round face**	+	+	-
**Valgus**	+	+	NA
**Hyperreflexia**	+	+	NA
**Simple ears**	+	+	-
**Cupped ears**	+	-	+
**Spastic quadriplegia**	+	+	-

MO→Moderate, S→Severe, NA→No data available,+→clinical feature present, -→clinical feature absent.

### Family D299

The index patient (III-3) is 16 years old and his two half-brothers (III-1, III-2) deceased at the ages of 21 years and 16 years respectively (Figure 1d). Clinical features are summarized in Table 1.

The three affected sons of family D299 had similar clinical features, characterized by severe psychomotor retardation, absent speech, seizures, hypotonia and contractures. The thyroid hormone profile of the index patient III-3 was T_3_-9 (Normal range: 2.6-5.7 pmol/L), T_4_-8.6 (Normal range: 9–19 pmol/L), TSH-2.16 (Normal range: 0.35-5 mU/L) and TGB-870 (Normal range: 233–490 nM/L). The patients have obtained thyroxin medication at the age of 2–3 years without any improvement.

X-exome sequencing of the index patient (III-3) revealed two novel variants, one in *SLC16A2* and the other in *ODZ1* (Additional file 1: Table S1). The 5 base-pair insertion identified in exon 3 of *SLC16A2* leads to a frameshift and a premature stop codon [NM_006517.4:c.990_991insGCTGC; p.G334PfsX11] and segregated with the clinical phenotype. The mother (II-2) carries the mutation on one of her X-chromosomes. The mother's sister (II-5) was also tested and she was found not to be a carrier. This novel mutation in the *SLC16A2* gene was absent in 100 Finnish controls.

### Family D174

**Figure 2:**
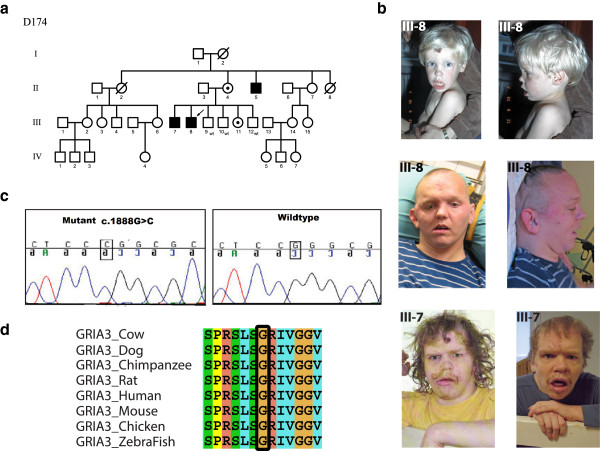
**Overview of the missense variant reported in *GRIA3 *in a Finnish Family with XLID. a)** Family pedigree showing the inheritance of the *GRIA3* mutation, open circles denote females; circles with a dot in the middle denote obligate carrier females, empty square denote males, the left half of the black squares denote affected males, the right half of the squares denote mutation positive males, crossed symbols denote deceased individuals, wt denote mutation negative subject, **b)** Photographs of the affected males, **c)** Sanger sequencing confirming the mutation, **d)** Multiple species protein sequence alignment showing conservation of the mutated G630 residue.

Family D174 has three affected males (III-7, III-8 and II-5, Figure 2a). The patients aged 36, 35 and 57 years respectively, are characterized by severe ID with autistic features, epilepsy, short stature and behavioral problems such as self injury and aggressive outbursts (Table 2).

**Table 2:** **Overview of clinical features of previously published patients with *GRIA3 * deletion/duplication and present patients with *GRIA3 *** **mutation**

	**Gecz et al. [1999] **[31]** Female patient**	**Wu et al. [2007] **[34]** Male patients**					**Bonnet et al. [2009] **[36]** Male/male patient**	**Chiyonobu et al. [2007] **[35]** Male patient**	**D174 Male patient (III-7)**	**D174 Male patient (III-8)**	**D174 Male patient (II-5)**
	**n = 1**	**n = 3**	**n = 2**	**n = 4**	**n = 2**	**n = 2**	**n = 2**	**n = 1**			
**Type of change**	Translocation t(X;12) (q24;q15)	Deletion	Missense variant p.G833R	Missense variant p.R631S	Missense variant p.M706T	Missense variant p.R450Q	Partial duplication of *GRIA3*	Partial duplication of *GRIA3*	Missense variant p.G630R	Missense variant p.G630R	Missense variant p.G630R
**Feature**											
**Intellectual disability**	+	+	+	+	+	-	+/+	+	+	+	+
**Mild**	-	-	-	-	+	-	-	-	-	-	-
**Moderate**	+	+	+	+	+	-	-	-	-	-	-
**Severe**	-	-	-	-	-	-	+/+	+	+	+	+
**Autistic features**	ND	ND	+	ND	ND	ND	+/ND	+	+	-	+
**Behaviour troubles**	+	ND	+	ND	ND	ND	+/+	+	+	+	+
**Self injury**	ND	ND	ND	ND	ND	ND	ND	ND	+	+	+
**Aggressive outbursts**	ND	ND	ND	ND	ND	ND	ND	ND	+	+	-
**Dysmorphic features**	ND	ND	ND	ND	ND	ND	+/+	+	+	+	+
**Brachycephaly**	ND	ND	ND	ND	ND	ND	ND	ND	+	+	+
**Macrocephaly**	ND	ND	+	ND	ND	ND	ND	ND	-	-	-
**Deep set eyes**	ND	ND	ND	ND	ND	ND	ND	ND	+	+	+
**Aesthenic habitus**	ND	ND	ND	+	+	ND	ND	ND	-	-	-
**Myclonic jerks**	ND	ND	+	ND	ND	ND	ND	ND	ND	ND	ND
**Hyporeflexia**	ND	+	ND	ND	+	ND	ND	ND	ND	ND	ND
**Prominent supraorbital ridges**	ND	ND	ND	ND	ND	ND	ND	ND	+	+	+
**Short stature**	ND	ND	ND	ND	ND	ND	-/-	+	+	+	+
**Epilepsy**	+	ND	ND	ND	ND	ND	-/-	-	+	+	+
**Inguinal hernia**	-	ND	ND	ND	ND	ND	+/-	+	-	-	-
**Brain MRI abnormalities**	ND	ND	ND	ND	ND	ND	-/+	-	ND	ND	ND
**Bowel occlusions**	ND	ND	ND	ND	ND	ND	ND	ND	-	-	+
**Malposition of feet**	ND	ND	ND	ND	ND	ND	ND	ND	+	+	-
**Hydronephrosis**	ND	ND	ND	ND	ND	ND	ND	ND	+	ND	ND
**Ren arcuatus**	ND	ND	ND	ND	ND	ND	ND	ND	+	ND	ND

ND→No data, +→clinical trait present, -→clinical trait absent.

X-exome sequencing of the index patient III-8 revealed a novel missense mutation [NM_000828.4:c.1888G > C; p.Gly630Arg] in exon 12 of the *GRIA3* gene that segregated in the family (Additional file 1: Table S1). The mutation was absent in 135 Finnish controls. Using PolyPhen-2, SIFT, PhD-SNP, SNPS&GO and PANTHER, the mutation was predicted to be probably damaging. The ConSeq server also revealed that this residue is highly conserved with a score of 9. Missense changes in *OR13H1*, *GPR112* and *F8* did not segregate with the phenotype. The variants identified in *TAF1* and *LRCH2* segregated in the family. However, both variants turned out to be common single nucleotide variations as they were present in 3/88 Finnish control samples.

The wild type residue glycine of the p.Gly630Arg is neutral and the mutant residue arginine is positively charged. HOPE [21] predicted that this can lead to disturbances in the ionic interactions with the other transmembrane helices. The mutant residue is larger than the wild type residue, which can disturb either the contacts with the other transmembrane domains or with the lipid membrane. This can also affect the hydrophobic interactions within the core of the protein or with the membrane lipids. The wild type residue, glycine, is considered to be the most flexible of all residues and this flexibility might be vital for this protein’s function. It is predicted that this glycine residue at this position is needed to make a special backbone conformation or to facilitate movement of the protein.

### Family D172

**Figure 3:**
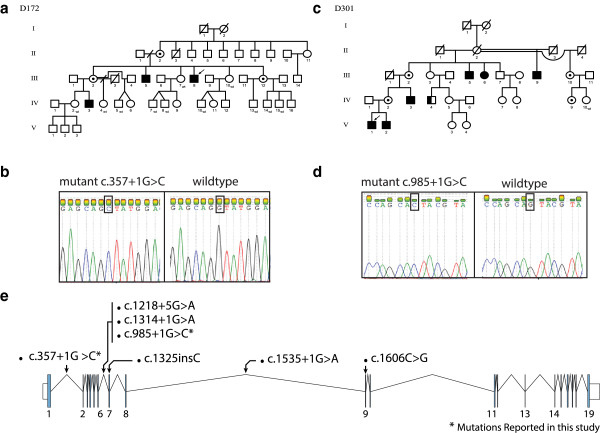
**Overview of the two splice donor mutations identified in *DLG3 *in two Finnish families. a)** Pedigree of family D172 showing the inheritance of the *DLG3* splice donor mutation c.357 + 1G > C, open circles denote females; circles with a dot in the middle denote obligate carrier females, empty square show males, the left half of the black squares denote affected males, the right half of the squares denote mutation positive males, crossed symbols denote deceased individuals, wt denote mutation negative subject, **b)** Sanger sequencing result showing the mutation in one affected male, **c)** Pedigree of family D301 showing the inheritance of the *DLG3* splice donor mutation c.985 + 1G > C, **d)** Sanger sequencing result showing the mutation in one affected male, **e)** Schematic representation of the *DLG3* gene, along with overview of previously reported mutations and mutations reported in the current study.

This family has three affected males in two generations (III-5, III-8 and IV-3, Figure 3a). X-exome sequencing of the index patient (III-8) revealed two novel variants, one in *DLG3* and the other in *ZBTB33* (Additional file 1: Table S1). The *ZBTB33* variant was excluded because it did not co-segregate with the phenotype. A donor splice site mutation [NM_021120.3:c.357 + 1G > C] in intron 1 of the *DLG3* gene segregates in the family. The affected males who presented with mild to moderate ID, normal growth, narrow thorax, molar hypoplasia, short up-slanting palpebral fissures, a high vaulted palate, and hypotonia. Daytime wetting has been a problem until adult age (Table 3). In addition to the aforementioned features the index patient (III-8) had an external strabismus in his right eye. Chromosomes (300bands), FRAXA, vacuolated lymphocytes, urinary metabolic screen, ophthalmology and hearing examinations showed normal results. Females II-2, III-2 and III-12 are heterozyous carriers of this mutation. Female III-2 has passed regular basic education and completed vocational schooling. Her younger son (IV-5) has epilepsy but is of normal intelligence and does not have the *DLG3* mutation. Female II-2 is 77 years old, who is also a mutation carrier and has no cognitive or memory problems. All females showed a normal X-inactivation pattern in their blood lymphocytes (data not shown).

**Table 3:** **Comparison of the clinical features of the patients of two families with *DLG3 *** **mutation**

	**Family**	**D172**		**Family D301**				
	**III-5**	**III-8**	**IV-3**	**III-5**	**III-9**	**IV-3**	**V-1**	**V-2**
	**Male**	**Male**	**Male**	**Male**	**Male**	**Male**	**Male**	**Male**
**Clinical features**								
**Intellectual disability**	+/MO	+/M	+/MO	+/S	+/S	+/MO	+/MO	+/MO
**Delayed motor development**	+	ND	+	+	ND	+	+	+
**Delayed speech development**	+	ND	+	+	+	+	+	+
**Eneuresis**	+	-	+	+	-	-	+	-
**Strabismus**	-	-	-	+	-	+	+	-
**ADHD**	-	-	-	ND	ND	+	+	+
**Seizures**	-	-	-	+	-	-	-	-
**Behavioural problems**	-	-	+	+	-	+	-	-

ND→No data, M→Mild, MO→Moderate, S→Severe, +→clinical feature present, -→clinical feature absent.

### Family D301

Family D301 has five affected males in three generations (III-5, III-9, IV-3, V-1 and V-2, Figure 3c). In the index patient, V-1, etiological investigations such as urine metabolic screening, karyotype analysis, Fragile-X testing, FISH on 22q11.2 locus, creatinine kinase, lactate, carnitine, very long fatty acids, EEG and brain MRI were normal.

The phenotype consists of delayed motor and language development. Three males had strabismus and attention deficit hyperactivity disorder (ADHD). The males do not have other dysmorphic features except a bifid uvula in patients V-1 and V-2. Only one patient had experienced seizures in childhood. The cognitive performance in the affected males varied from severe to moderate ID, with ID being less severe in the younger generations (Table 3). Female III-6 participated in the cognitive evaluation at the age of 60 years at the level of moderate ID; she had not been evaluated earlier. She had received her basic and vocational education in a special school. She has not been able to work but has lived independently and needs assistance in all paperwork and contacts with authorities. The mother (IV-2) of patients V-1 and V-2 and their grandmother (III-2) graduated from regular schools and obtained vocational education.

X-exome sequencing of the index patient (V-1) identified a novel donor splice site mutation [NM_021120.3:c.985 + 1G > C] in intron 6 of the *DLG3* gene (Additional file 1: Table S1). One affected carrier female (III-6) had a skewed X-inactivation pattern of 80:20 (Additional file 2: Figure S1).

### Family D222

**Figure 4:**
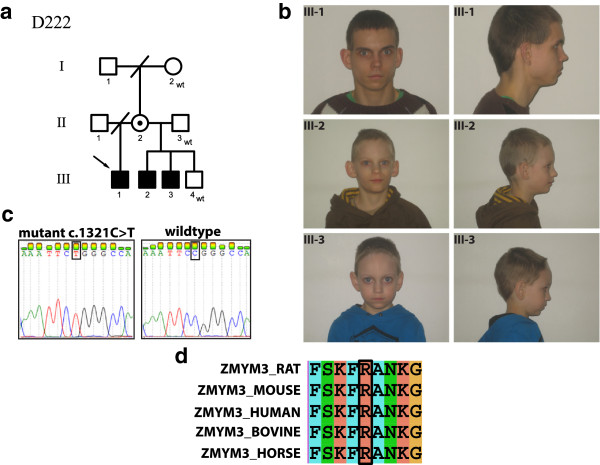
**Clinical presentation of a novel syndrome in a Finnish family with XLID and segregation of the *ZMYM3 *missense mutation. a)** Pedigree showing the inheritance of the *ZMYM3* mutation in family D222, open circles show females, circles with a dot in the middle show obligate carrier females, empty square show males, the left half of the black squares show affected males, the right half of the squares show mutation positive males, crossed symbols denote deceased individuals, wt denote mutation negative subject, **b)** Photographs of the three affected males, **c)** Sanger sequencing confirming the mutation, **d)** Multiple species protein alignment showing conservation of the mutated R441 residue in ZMYM3.

Family D222 has three affected males who are half-brothers (III-1, III-2 and III-3, Figure 4a). The phenotype consists of small gestational age (SGA), hypospadia, mild aortic stenosis and leakage of the aortic valve, arcus aortae dexter, horseshoe kidney, slenderness and cup formed ear lobes. Their motor and language development were slightly delayed and they continue to have nocturnal enuresis up to school age. Two of them have ADHD. The cognitive performance is at the level of mild ID. The development of the youngest brother (III-4) is normal (Table 4).

**Table 4:** **Summary of clinical findings of patients with the *ZMYM3 *** **mutation**

**Clinical trait**	**Patient III-1**	**Patient III-2**	**Patient III-3**
**Age**	15.5 y	8.8 y	7.3y
**Weight at birth**	2530 g	2460 g	3590 g
**Head circumference**	55.5 cm, −1 SD	49.6 cm, −3 SD	49.5 cm, −2.7 SD
**Hypospadia**	+	+	-
**Horseshoe kidney**	-	+	-
**Enuresis nocturna**	+	+	rarely
**Large cupped formed ear lobes**	+	+	+
**ADHD**	+	+	ND
**Arcus aortae dexter**	-	+	-
**Bicuspid aortic valve**	+	+	-
**Sleep disorder**	+	+	+
**Intellectual disability**	+/M	+/M	+/M

M→Moderate, +→clinical trait present, -→clinical trait absent, SD→standard deviation.

X-exome sequencing of the index patient (III-1) identified novel variants in *PTCHD1*, *RAB40A*, *ABCD1*, *COL4A5* and *ZMYM3*, respectively (Additional file 1: Table S1). *PTCHD1* and *RAB40A* were excluded as a candidate gene because their variants did not segregate in the family (Additional file 1: Table S1). We suggest that the variant in *COL4A5* is unlikely pathogenic because mutations in this gene have been previously associated with Alport syndrome [OMIM#301050].

The variant in *ABCD1* was excluded as a causative mutation as mutations in *ABCD1* previously have been linked to adrenoleukodystrophy [OMIM#300100]. The novel missense mutation [NM_201599.2:c.1321C > T; p.Arg441Trp] in exon 7 of the *ZMYM3* gene was present in all three affected males and absent in their healthy brother. The mother (II-2) was found to be a carrier of this mutation. This mutation leads to substitution of the amino acid arginine with tryptophan (p.Arg441Trp) and was absent in 100 Finnish anonymous blood donors.

PolyPhen-2, SIFT, PhD-SNP, SNPS&GO and PANTHER predicted this mutation to be damaging. The ConSeq server also revealed that this residue is highly conserved with a score of 9. HOPE [21] predicted that the mutant residue is larger and more hydrophobic than the wild type residue. The amino acid substitution will lead to loss of hydrogen bonds in the core of the protein, thus preventing correct folding. The difference in the amino acid properties is likely to disturb a Zinc-finger domain known to bind DNA.

### Family D175

**Figure 5:**
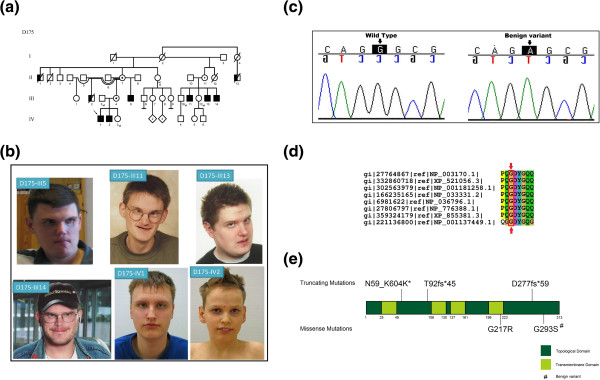
**A clinical presentation of a novel syndrome in a Finnish family (D175) with XLID. a)** Family pedigree showing the inheritance of the benign variant in *SYP*, open circles denote females, circles with a dot in the middle denote obligate carrier females, empty square denote males, the left half of the black squares denote affected males, the right half of the squares denote mutation positive males, crossed symbols denote deceased individuals, wt denote mutation negative subject, **b)** Photographs of the six affected males, **c)** Sanger sequencing confirming the benign variant, **d)** Multiple species protein alignment showing conservation of the mutated Gly293 residue in SYP, **e)** Schematic presentation of the SYP protein domains, location of the published mutations and the polymorphism identified in this study.

This family has 9 affected males in three generations (II-1, II-13, III-2, III-5, III-11, III-13, III-14, IV-1 and IV-2, Figure 5a). The affected males are characterized by mild to moderate ID, normal growth, and dysmorphic facial features with prominent supraorbital ridges, deep set eyes, short philtrum, and prominent chin suggesting a novel syndrome (Figure 5b). One patient has epilepsy (III-5). Patients III-5, IV-1 and IV-2 have mild brain abnormalities in MRI (corpus callosum hypoplasia, mild cortical atrophy). Only limited clinical data is available for patients III-11, III-13 and III-14.

X-exome sequencing of the index patient (IV-1) identified a recurrent missense variant in exon 6 of the *SYP* gene [NM_003179.2:c.877G > A; p.Gly293Ser]. This missense variant was found in all the affected males. Variants in *DMD*, *FAM47A*, *USP9X* variants were not found in all of the affected males (Additional file 1: Table S1). Variant in *FAM47B* was not analyzed.

## Discussion

In six families we identified five pathogenic mutations in three known XLID genes (*SLC16A2*, *GRIA3* and *DLG3*) and one in the candidate XLID gene *ZMYM3*. In addition, two novel syndromes were identified, one with a novel missense mutation in a candidate XLID gene, *ZMYM3*[23] and the other with a previously reported benign variant in *SYP* in a large family (D175).

We identified two pathogenic mutations in *SLC16A2*. Mutations in this gene are known to cause Allan-Herndon-Dudley syndrome (AHDS; OMIM #300523). AHDS is typically characterized by developmental delay, poor head control, poor or no speech and muscle hypoplasia [24]. The clinical features of the patients investigated in this study are characteristic of AHDS. Previously, mutations in the *SLC16A2* gene have been reported in more than 45 families across the world and one *de novo* translocation t(X;9)(q13.2;p24) has been reported in a female patient [24-29]. *SLC16A2* belongs to the *SLC16* gene family [30] and encodes the monocarboxylate transporter 8 (MCT8) protein, which is expressed in brain, liver, heart, intestine, placenta, kidney and thyroid. In the central nervous system MCT8 is present in neurons and astrocytes and is an essential thyroid hormone transporter, involved in the transport of thyroid hormone (TH) across the blood–brain barrier. The *SLC16A2* gene consists of six exons and encodes a protein of 539 amino acids [24]. MCT8 contains twelve transmembrane domains (TMD) with intracellular amino- and carboxy-terminal domains [24]. *In vitro* studies showed that mutations in this gene lead to a reduced or absent supply of triiodothyronine (T3) to neurons [30]. The novel frameshift mutation p.G334PfsX11 identified in family D299 is located in the seventh TMD and the recurrent p.Arg371Cys amino acid substitution present in family L107 lies in the eighth TMD. The same missense mutation has previously been identified in a sporadic case (annotated as c.1333C > T, p.Arg445Cys using a different SLC16A2 isoform [29]. It has been previously shown that in a moderately affected individual with c.1333C > T missense mutation resulted in a more severe decrease in T3 uptake [29]. In the article by Capri et al. [29], the affected patient, aged 13 years, is able to walk with aid and can speak a few words. In family L107 the phenotype is variable as the two oldest patients (II-1 and II-2) never walked whereas the youngest II-9 could walk until early childhood. The patients in family L107 could speak some words. Based on the available data p.Arg445Cys mutation seems to be associated with variable phenotypes. To our knowledge, these are the first families with AHDS in the Finnish population.

We identified a missense mutation c.1888G > C (p.Gly630Arg) (RefSeq NM_000828.4) in the glutamate receptor, ionotropic, AMPA 3 (*GRIA3*) gene. *GRIA3* belongs to a class of an alpha-amino-3-hydroxy-5-methyl-4-isoxazole propionate (AMPA)-sensitive *g*lutamate receptor that operates as a ligand-gated ion channel in the central nervous system and has an essential role in excitatory synaptic transmission [31]. These receptors contain three functional domains – transmembrane, ligand binding, and receptor channel core [32]. Ionotropic glutamate receptors (iGluRs) are related to learning and memory [33]. *In vitro* functional studies on *GRIA3* missense variants have shown that mutations in functional domains of *GRIA3* are associated with kinetic changes in AMPA receptor function leading to significant reduction in iGluR3 channel function which is found to be linked with moderate ID [34,35]. Previously, four missense mutations, one whole gene deletion and three duplications have been reported in *GRIA3*[34-38]. One of the missense mutations (p.Arg631Ser) [34] affected the amino acid residue adjacent to the Gly630 mutated in the family described here. The patients of these families have facial dysmorphic features in common and these are different from the other reported patients with a mutation in *GRIA3*. The affected individuals of family D174 exhibit a severe phenotype. They are all severely intellectually disabled with behavioral disturbances. Three of the four previously reported patients with a *GRIA3* mutation were moderately intellectually disabled, whereas one patient with a missense change (p.Met706Thr) had mild ID [34]. We conclude that there is wide variation in the severity of ID among patients with mutations in the *GRIA3* gene. Further phenotype studies are needed to confirm the syndromic features underlying *GRIA3* mutations.

The *DLG3* gene, located at Xq13.1, encodes the synapse-associated protein SAP102 [39]. SAP102 is a member of the membrane-associated guanylate kinase (MAGUK) protein family [39,40]. SAP102 is highly expressed in both young and mature neurons and localizes to the postsynaptic density of excitatory synapses [41]. It is the first XLID gene to be associated to glutamate receptor-mediated postsynaptic signaling, a process which is crucial for the regulation of synaptic formation and plasticity in brain development [40]. Mutated *DLG3* has been identified as a rare cause of XLID with, so far, only five families diagnosed, with a total of 17 affected males presenting with moderate to severe ID [41,42]. Of eleven mutation carrier women, one has mild ID and in another family a female carrier has mild ID and a history of seizures. These expressing females, however, do not have a skewed X-inactivation pattern [41,42]. Our families expand the phenotype spectrum of *DLG3* mutations to mild ID.

The missense mutation c.1321C > T (p.Arg441Trp) (RefSeq NM_201599.2) is the first variant in the coding region of *ZMYM3* reported to date. Previously, a chromosomal breakpoint in the 5’ UTR of *ZMYM3* has been reported in an intellectually disabled female. In addition to ID, the patient had scoliosis and spotty hyperpigmentation of the skin. She also had slight facial asymmetry and clinodactyly [23]. However, the patients described in this study do not have any of the reported clinical features except for mild ID. Recently, a missense variant of unknown significance (c.356A > G (p.Gln119Arg)) in *ZMYM3* was reported in the index patient of a family with XLID with four affected males in two generations. However, the patient also carries a missense change in the established XLID gene *HCFC1*[43].

The *ZMYM3* gene is also referred to as *ZNF261* and *DXS6673E. ZMYM3* belongs to the MYM family. This gene contains 5 tandem repeats of a Cys-X_2_-Cys-X_19–22_-Cys-X_3_-Cys-X_3_-Cys-X_13–19_-Cys-X_2_-Cys-X_19–25_-Cys-X_3_-Cys motif [44]. *ZMYM3* encodes a protein that is an integral component of histone deacetylase-containing multiprotein complexes. Further clarification is needed regarding the role of *ZMYM3* as a XLID gene [45]. In addition, a previously reported benign polymorphism in *SYP* was identified in six affected males with a syndromic phenotype (Additional file 1: Table S1). Pathogenic mutations in *SYP*, including three truncating and one missense change (OMIM **#**300802) have previously been reported [11]. The patients in our study presented with mild to moderate ID, epilepsy, and common dysmorphic features. The missense benign variant c.877G > A (p.Gly293Ser) (RefSeq NM_003179.2) has been reported in the Exome Variant Server with a MAF frequency of T = 0.001/1 suggesting that *SYP* is not explaining the syndromic phenotype in this large family.

The diagnostic yield based on our current study of 14 families is 35%, of which five pathogenic mutations are likely to be disease causing. The role of *ZMYM3* as a XLID gene is still questionable and can further be substantiated by the identification of additional families and mutations.

In eight families, work is ongoing as a functionally relevant mutation has not yet been identified. This might be due to technical limitations or a non-X-chromosomal origin of the disorders. In five of the mutation “negative” families the affected are brother pairs, in which linkage is less strong than for the other families.

## Conclusions

The first X-exome sequencing study of Finnish families with intellectual disability revealed the first two Finnish families with Allan-Herndon-Dudley syndrome, one family with a novel mutation in *GRIA3*, and two families with novel mutations in *DLG3.* In addition, a novel missense mutation in the candidate XLID gene *ZMYM3* cosegregated with a novel syndrome. A seventh family was found to have a benign missense variant *SYP* and a syndromic phenotype. Further studies are needed to find the causative mutation in this extended family.

This study demonstrates the power of exome sequencing in finding rare mutations underlying intellectual disability. It has been estimated that roughly 10% of the protein coding genes causing ID are located on the X-chromosome. Based on our research results, the likelihood of finding a genetic cause for the disease is increased in families with several affected males. We hypothesize that in some of the families, rare autosomal recessive mutations may be identified due to the characteristics of the founder population of Finland [46].

### Web resources

The URLs for data presented are as follows:

dbSNP, http://www.ncbi.nlm.nih.gov/SNP/

ConSeq, http://conseq.tau.ac.il/

PolyPhen-2, genetics.bwh.harvard.edu/pph2/

SIFT, sift.jcvi.org

Phd-SNP, http://snps.biofold.org/phd-snp/phd-snp.html

Refseq, http://www.ncbi.nlm.nih.gov/refseq/

SNPs &GO, http://snps.biofold.org/snps-and-go/snps-and-go.html

PANTHER, http://www.pantherdb.org/tools/csnpScoreForm.jsp?

HOPE, http://www.cmbi.ru.nl/hope/input

MERAP, http://www.sourceforge.net/projects/merap/files/MERAP20131101/

OMIM, http://www.ncbi.nlm.nih.gov/omim/

NHLBI Exome Sequencing Project (ESP)

Exome Variant Server, http://evs.gs.washington.edu/EVS/

## Abbreviations

XLID: X linked intellectual disability; MCT8: Monocarboxylate transporter 8; ADHD: Attention deficit hyperactivity disorder; AHDS: Allan-Herndon-Dudley syndrome.

## Competing interests

We declare that we have no competing interest.

## Authors’ contributions

IJ conceived the idea of the study. AS, KA, MS, HK, MP and MA were responsible for the clinical diagnosis of the patients. AKP designed the experiments, performed mutation analysis for the families, X-inactivation study and wrote the manuscript. HVE and GF analyzed family L107. FD and MAh were involved in the mutation analysis study of the family D174 and family D172. SAH, HU and VMK performed X-exome sequencing and bioinformatic analysis. All authors contributed to the final manuscript version.

## Supplementary Material

Additional file 1: Table S1 List of all the variants identified using X-exome sequencing. Click here for file

Additional file 2: Figure S1 X inactivation pattern of an affected carrier female III-6 in Family D301. Click here for file
